# Letter to the editor: Atypical weather is associated with the 2022 early start of West Nile virus transmission in Italy

**DOI:** 10.2807/1560-7917.ES.2022.27.34.2200662

**Published:** 2022-08-25

**Authors:** José Lourenço, Francesco Pinotti, Taishi Nakase, Marta Giovanetti, Uri Obolski

**Affiliations:** 1 Biosystems and Integrative Sciences Institute, Faculty of Sciences, University of Lisbon, Lisbon, Portugal; 2 Department of Zoology, University of Oxford, Oxford, United Kingdom; 3 Nuffield Department of Medicine, University of Oxford, Oxford, United Kingdom; 4 Department of Science and Technology for Humans and the Environment, University of Campus Bio-Medico di Roma, Rome, Italy; 5 Laboratório de Flavivírus, Instituto Oswaldo Cruz, Fundação Oswaldo Cruz, Rio de Janeiro, Brazil; 6 School of Public Health, Faculty of Medicine, Tel Aviv University, Tel Aviv, Israel; 7 Porter School of the Environment and Earth Sciences, Faculty of Exact Sciences, Tel Aviv University, Tel Aviv, Israel

**Keywords:** West Nile virus, surveillance, climate change, One Health, transmission suitability

**To the editor: **In the recent study by Barzon et al. [1], the authors report an unusually early onset of West Nile virus (WNV) transmission in Italy in 2022. The virus was detected in mosquitoes on 8 June and in humans on 18 June, about one month earlier than usually detected. In addition to describing the epidemiological and genetic characteristics of cases, Barzon et al. suggest that the early WNV season may be due to changes in weather conditions, mentioning that March–May 2022 has been an unusually dry and hot period in northern Italy.

Transmission suitability indices are commonly used to investigate the relationships between weather and mosquito-borne disease. We have specifically developed [2] and regularly apply such a transmission suitability measure to investigate the spread of WNV spread, e.g. in Portugal, Israel and Brazil [3-5]. As we regularly estimate WNV transmission suitability across Europe, we have recently noted that our early estimates for Italy during 2022 align with the important observations made by Barzon et al. in their paper.

In Italy, there is typically a steady increase in estimated WNV transmission suitability between April and July. Accordingly, the first reported cases appear during mid-July or later. In line with the hypothesis by Barzon et al., we noticed that May and June 2022 exhibited unusually high estimated transmission suitability (https://doi.org/10.6084/m9.figshare.20489151.v1). These estimates were second only to 2018 – the year in which the largest WNV outbreak of the last decade occurred in Italy. Underlying the observed increases in suitability in 2022 was a country-wide average increase in temperature of ca 2 and 2.7^◦^C in May and June, respectively, compared with the mean of the past 10 years. In parallel, there was a tendency for dryer weather, with average decreases of 3.9 and 6.5% in relative humidity in May and June 2022, respectively. This supports the association between the atypical weather conditions and the early WNV transmission season in Italy during 2022, as was hypothesised by Barzon et al.

Variation in local weather is long known to be a major factor affecting the dynamics and spread of mosquito-borne diseases. Suitability measures informed by climate data cannot only retrospectively estimate the transmission potential of a virus, but can also provide real-time insights on the start and potential magnitude of current transmission seasons. Such insights are becoming increasingly important, not only to inform policymakers but to help mitigate future outbreaks in the context of ongoing climate change trends. As Europe experiences an ever-increasing reporting rate of local transmission events of mosquito-borne viruses, additional resources should be invested in transmission suitability estimation methodologies, in parallel with enhancing surveillance infrastructure and research of these pathogens.
